# Effect of Quanzhenyiqitang on apoptosis of alveolar macrophages and expression of histone deacetylase 2 in rats with chronic obstructive pulmonary disease

**DOI:** 10.3892/etm.2014.1585

**Published:** 2014-02-25

**Authors:** DA-ZHI LI, SHI-WEI RUAN, ZHI-BIN CHEN, CHUN-E. WANG

**Affiliations:** 1 Fujian University of Traditional Chinese Medicine, Fuzhou, Fujian 350000, P.R. China; 2 Department of Respiration, The Second Affiliated People’s Hospital of Fujian University of Traditional Chinese Medicine, Fuzhou, Fujian 350000, P.R. China; 3 Medical Center of the Affiliated People’s Hospital of Fujian University of Traditional Chinese Medicine, Fuzhou, Fujian 350000, P.R. China; 4 The Fujian Research Academy of Traditional Chinese Medicine, Fuzhou, Fujian 350000, P.R. China

**Keywords:** chronic obstructive pulmonary disease, alveolar macrophage, Quanzhenyiqitang

## Abstract

This study aimed to investigate the effect of Quanzhenyiqitang on alveolar macrophages (AMs) in a rat model of chronic obstructive pulmonary disease (COPD). In addition, the induction of apoptosis and regulation of histone deacetylase 2 (HDAC2) was studied to elucidate the underlying mechanisms of Quanzhenyiqitang treatment of COPD. Quanzhenyiqitang-treated serum was applied to AMs obtained from rats with COPD. A blank (control) group, an untreated serum group and an aminophylline group were also observed to evaluate the differences in AM apoptosis status, as well as the expression levels of caspase-9, caspase-8 and HDAC2. Compared with the control group, Quanzhenyiqitang-treated serum resulted in higher levels of caspase-9 and caspase-8 expression, increased apoptosis of AMs and increased expression of HDAC2 by AMs. In conclusion, Quanzhenyiqitang is capable of inducing apoptosis of AMs, which are the primary inflammatory cells in COPD, and modulating the expression of the important inflammatory factor HDAC2, producing an overall anti-inflammatory effect.

## Introduction

Chronic obstructive pulmonary disease (COPD) is a systemic inflammatory disease that primarily affects the lungs. It is an important disease with high incidence and mortality rates worldwide. Chronic inflammation is its basic pathology and the key cause of morbidity (1). At present, global guidelines for the management of COPD using Western medicine include treatment with bronchodilators and oral or inhaled corticosteroids to improve ventilation and alleviate airway inflammation. Antibiotic treatment is recommended for infection control in the acute phase, as well as the administration of Pneumococcal vaccine or influenza vaccine, such as the H1N1 vaccine, in remission to reduce the recurrence of the disease (2). However, these methods are associated with several problems, particularly in relation to the systemic effects of COPD, including poor symptom control, frequent relapse and adverse drug reactions (3). Furthermore, there has been a lack of focus on the resolution of these problems. For patients with COPD, the systemic effects of the disease cause progressive deterioration, including loss of appetite, severe malnutrition and osteoporosis (4), and there are few effective treatments available. Thus, the quality of life of the patients becomes seriously diminished and disease prognosis worsens.

Quanzhenyiqitang has been used to treat the systemic effects of COPD. A study demonstrated that Quanzhenyiqitang was capable of improving clinical signs and symptoms of patients with chronic obstructive pulmonary emphysema (5). An experiment using a rat model of COPD also indicated that Quanzhenyiqitang was capable of significantly improving the cell morphology of damaged lung, kidney, adrenal gland and testicular tissue (6). However, the mechanism by which Quanzhenyiqitang improves damaged tissue has yet to be elucidated. In this study, rat alveolar macrophages (AMs) were treated with Quanzhenyiqitang-treated serum, and the ability of Quanzhenyiqitang to induce apoptosis of inflammatory cells and to modulate the expression of histone deacetylase 2 (HDAC2) was evaluated. The anti-inflammatory effect and therapeutic mechanisms of Quanzhenyiqitang in the treatment of COPD are discussed.

## Materials and methods

### Animals

Male 42-month-old Sprague Dawley rats (weight, 200±5 g) were provided by the Experimental Animal Center of B&K Universal Group Limited (Shanghai, China). The rats were reared in the animal housing facility of the Fujian Research Academy of Traditional Chinese Medicine (Fuzhou, China) and were fed with standard grain in the clean facilities of the animal room in Pingshan College of the Fujian University of Traditional Chinese Medicine (Fuzhou, China). Acclimation was performed for seven days under the following conditions: free water and food, room temperature of 20°C and natural light. Among the rats, 30 (of a total of 40) were used for treated serum preparation; the others were used as rat models of COPD.

### Rat model of COPD

The rat model of COPD was prepared according to the methodology of the Respiratory Department of Shuguang Hospital Affiliated to Shanghai University of Traditional Chinese Medicine (Shanghai, China) and combined with kidney deficiency (7). Rats were fed daily with 1% adenine granulated feed (obtained from the Medical Research Institute of Fujian Province, Fuzhou, China), provided with normal drinking water, and subjected to smoke treatment with 250 ppm SO_2_ (Tianjin Specialty Gases Co., Ltd., Tianjin, China) for 5 h/day, 5 days/week for 7 weeks. This study was conducted in accordance with the Guide for the Care and Use of Laboratory Animals of the National Institutes of Health. The animal use protocol was approved by the Institutional Animal Care and Use Committee of the Second Affiliated People’s Hospital of Fujian University of Traditional Chinese Medicine (Fuzhou, China).

### Grouping

Cultured AMs were randomly divided into four groups as follows: Blank (group A), untreated serum (group B), serum treated with Traditional Chinese Medicine (Quanzhenyiqitang; group C), and serum treated with Western medicine (aminophylline; group D).

### Cell culture

Rats were anesthetized by intraperitoneal injection of pentobarbital sodium (30 mg/kg body weight). The chest of each rat was opened, and the right side of the main bronchus was tied. The left lung was then lavaged four times with 5 ml saline solution at 37°C, and the alveolar lavage fluid was collected and centrifuged at 106 × g for 10 min at 4°C. The supernatant was subsequently discarded, and the pellet was washed twice with phosphate-buffered saline (PBS) for 10 min each time. The cells were resuspended in low-sugar Dulbecco’s modified Eagle medium (DMEM) with fetal bovine serum and counted. Cell viability was measured to be >98%. The cells were then replated in six-well plates (1 ml/well; 1×10^9^ cells/l) and cultured at 37°C in 5% CO_2_ for 2 h. Non-adherent cells were removed by washing with PBS. Low-sugar, serum-free DMEM (1 ml) and macrophage stimulating protein (50 μl) were added, prior to the cells being incubated for 24 h and centrifuged at 45 × g for 10 min. The cell culture supernatant was then collected.

### Preparation of treated serum

A total of 30 rats were randomly and equally divided into three groups. The untreated serum group was lavaged four times with 5 ml saline solution. The Traditional Chinese Medicine group was lavaged with Quanzhenyiqitang (15 g stewed, sun-cured ginseng, 15 g radix ophiopogonis, 15 g cooked rehmannia, 6 g light monkshood that had been decocted for 20 min, 6 g Atractylodes, 15 g Achyranthes and 6 g Schisandra; Fuzhou Tongchun Medicine Co., Ltd., Fuzhou, China). The Western medicine group was lavaged with aminophylline (0.25 g/2 ml; batch no. 110402; Shanghai Xinyi Jinzhu Pharmaceutical Co., Ltd., Shanghai, China) twice every day. All groups were lavaged twice every day.

The rats were lavaged for seven days. On the seventh day, the rats underwent a 12-h fast and were then lavaged once with a full daily dose. At 1 h post-dose, the rats were anesthetized by intraperitoneal injection of pentobarbital sodium (0.2 ml/100 g). Following routine disinfection, 6 ml abdominal aortic blood was collected from each rat and transferred to a negative-pressure vessel using a puncture needle in aseptic conditions. The blood samples were then placed in a water bath at 37°C for 15 min and centrifuged at 402 × g for 15 min. The serum was filtered through a 0.22-μm microporous membrane and the cells were moved to new Eppendorf tubes and stored at −20°C. The treated serum and serum-free culture medium were combined in a proportion of 1:4 to produce a new culture medium containing 20% treated serum.

### Observation of apoptosis

The AM culture medium was purified and the cells were cultured for 4 h. Fresh cell culture fluid was added and the cell culture wells were divided into four experimental groups, comprising a blank group and groups treated with untreated serum, 100 μl Quanzhenyiqitang-treated serum and 100 μl aminophylline-treated serum, respectively. Following culture at 37°C with 5% CO_2_, the cells were harvested at 2, 4 and 6 h. Upon staining with an Annexin V-fluorescein isothiocyanate/propidium iodide kit (Viaud Co. Ltd., Shanghai, China), in accordance with the manufacturer’s instructions, apoptotic cell morphology was observed under a fluorescence microscope (Olypus, Tokyo, Japan). Flow cytometry was performed to determine the rate of apoptosis of AMs at the 2-, 4- and 6-h time-points.

### Fluorescence quantitative polymerase chain reaction (PCR)

In the previous experiment, apoptosis was observed at the 6-h time-point. Therefore, cells harvested at the 6-h time-point were used to assess gene expression. The cells were washed twice with PBS, centrifuged and resuspended in TRIzol^®^ (Invitrogen Life Technologies, Carlsbad, CA, USA), prior to being left to rest for 5 min at room temperature. Chloroform (~200 μl) was added and the suspension was then rested again at room temperature for 5 min. The cells were subsequently centrifuged at 8,765 × g for 20 min at 4°C, the supernatant was collected and an equal volume of isopropyl alcohol was added, blended and precipitated at 80°C for 1 h.

Following centrifugation at 8,765 × g for 20 min at 4°C, the supernatant was discarded, 75% ethanol (~800 μl) was added and the pellet was washed and precipitated. A further round of centrifugation (8,765 × g for 20 min at 4°C) was performed, 75% ethanol (800–1,000 μl) was added and the pellet was washed and precipitated. Following a final round of centrifugation, the supernatant was discarded, the pellet was air-dried and diethylpyrocarbonate (DEPC)-water was added at an appropriate volume until the precipitate was completely dissolved. The suspension was cryopreserved at −70°C, and the samples were used for electrophoretic and ultraviolet analyses.

A total of 1–4 μg total RNA was transferred to a 0.2-ml PCR tube, and 1 μl random primers and 12 μl DEPC-water were added. The solution was vortexed and placed in a −70°C ice bath for 3–5 min. Approximately 4 μl 5X reaction buffer was added to make a final volume of 20 μl. Following mixing, the solution was placed in a 42°C water bath for 60 min, a 70°C water bath for 10 min and then stored at 20°C. The reverse transcription products were subjected to fluorescence quantitative PCR. The 96-well PCR plate was covered with a sealing membrane (exclusively used in fluorescence quantitative PCR; Bio-Rad, Hercules, CA, USA), centrifuged and placed in a quantitative PCR machine (Bio-Rad). Data were then collected and analyzed.

### Western blotting

Confluent AMs were taken from the serum-free M199 culture solution and cultivated for 24 h. The cells were added to their respective culture media, blank serum and treated serum, and cultured for 24 h until the cells were synchronized at G_0_. The supernatant was discarded, and the pellet was washed three times with cold PBS and air-dried. A specific volume of cell lysates was added to the cell pellet for a few minutes. Using a cell scraper, the cells were transferred to a centrifuge tube along with the cellular debris and pyrolysis liquid. Centrifugation was performed at 6,440 × g for 10 min at 4°C. The precipitate was discarded, and the supernatant was immediately placed in a new centrifuge tube and stored at −20°C.

A sample of the supernatant protein was assessed using a bicinchoninic acid protein assay kit (Viaud Co. Ltd.). According to the optical density value and the standard concentration of protein, the standard curve was drawn and the total protein concentration of the samples was calculated.

The supernatant was combined with an epoxy potting compound, and electrophoresis, protein transfer and immune detection were performed. The supernatant was sensitized, developed and fixed via X-ray film photography in the darkroom. All reagents were provided by Shanghai Weiao Co., Ltd (Shanghai, China).

### Statistical analysis

Data were analyzed using the SPSS 17.0 statistical software package (SPSS, Inc., Chicago, IL, USA). Results are expressed as the mean ± standard deviation, and one-way analysis of variance was performed once variance was confirmed to be equal (P<0.05) using Levene’s test. Multiple comparisons between groups were analyzed by the least significant difference t-test. Comparisons between two means were analyzed using the Student-Newman-Keuls q-test. P<0.05 was considered to indicate a statistically significant difference.

## Results

### Cell apoptosis

**Figure 1:**
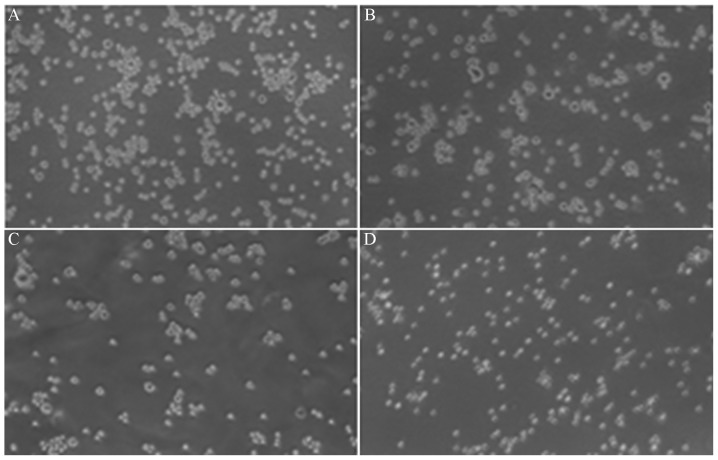
Microscope images of apoptosis of alveolar macrophages derived from rats with chronic obstructive pulmonary disease. (A) Blank group; (B) untreated serum group; (C) Quanzhenyiqitang-treated serum group; (D) aminophylline-treated serum group. Magnification, ×100.

**Figure 2:**
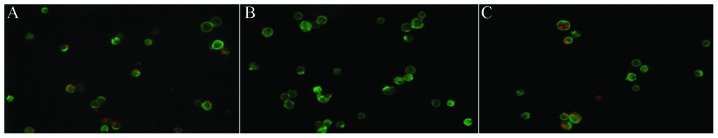
Apoptotic cell morphology under the fluorescence microscope. (A) Untreated serum group; (B) Quanzhenyiqitang-treated serum group; (C) aminophylline-treated serum group. Magnification, ×200.

**Figure 3:**
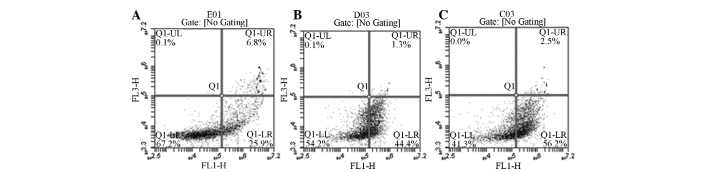
Flow cytometry of apoptotic alveolar macrophages derived from rats with chronic obstructive pulmonary disease (COPD), harvested after 6 h of cell culture. (A) Untreated serum group; (B) Quanzhenyiqitang-treated serum group; (C) aminophylline-treated serum group.

Microscopic observations and the analysis of the rate of apoptosis using flow cytometry demonstrated that Quanzhenyiqitang-treated serum resulted in a significantly higher rate of AM apoptosis compared with the other three experimental groups in the rat model of COPD (Figs. 1–3).

### Fluorescence quantitative PCR

**Figure 4:**
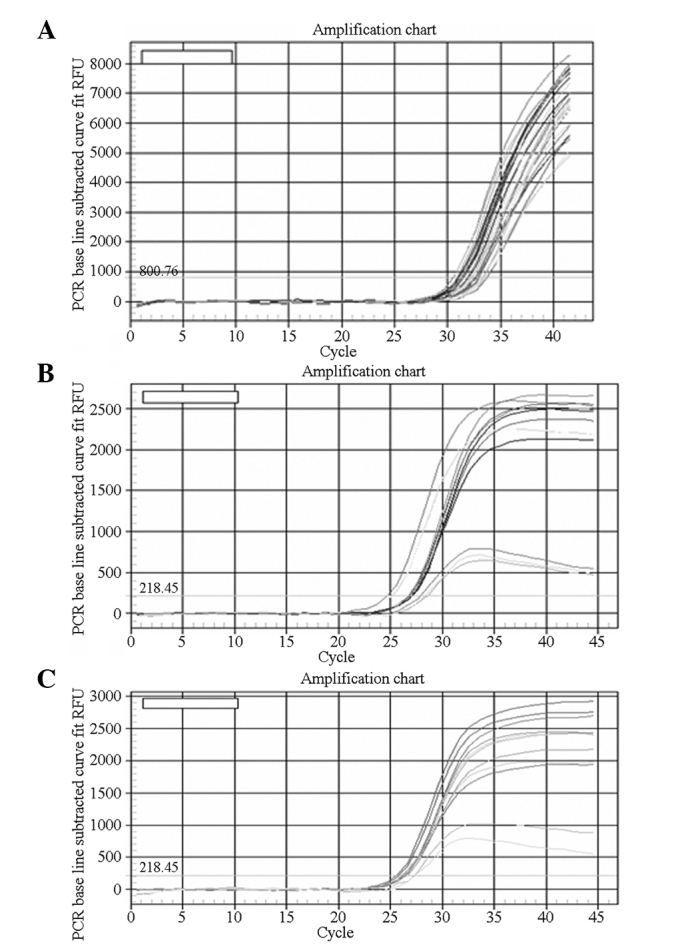
Fluorescence quantitative PCR. (A) Caspase-9; (B) caspase-8; (C) histone deacetylase 2. PCR, polymerase chain reaction; RFU, relative fluorescence units.

Caspase-8 gene expression was significantly higher in AMs of the rats of the Quanzhenyiqitang-treated serum group compared with the other three experimental groups. Levels of caspase-9 gene expression were similar in the Quanzhenyiqitang- and aminophylline-treated serum groups, which were higher than in the control and blank serum groups. Fluorescence quantitative PCR also demonstrated that HDAC2 gene expression was significantly higher in AMs in the rats of the Quanzhenyiqitang-treated serum group compared with the other three groups (Fig. 4).

### Western blotting

**Figure 5:**
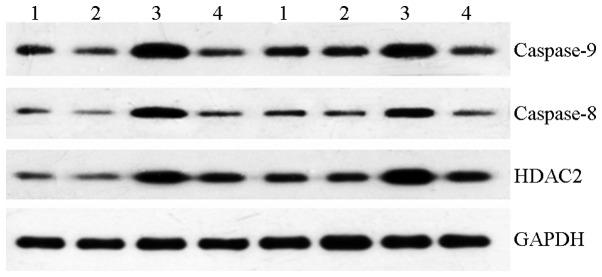
Protein levels of caspase-8, caspase-9 and HDAC2 in alveolar macrophages from rats with chronic obstructive pulmonary disease examined using western blotting. Lane 1: Blank group; Lane 2: untreated serum group; Lane 3: Quanzhenyiqitang-treated serum group; Lane 4: aminophylline-treated serum group.

**Figure 6:**
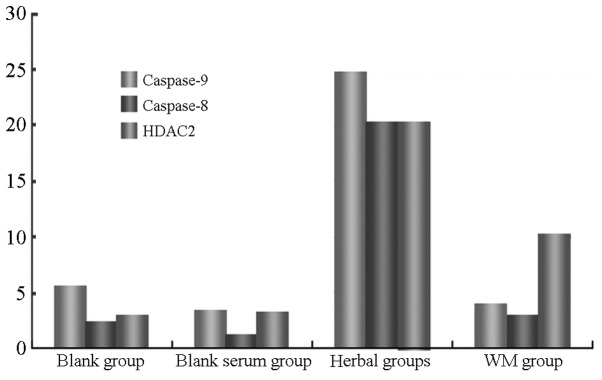
Protein levels of caspase-8, caspase-9 and HDAC2 in alveolar macrophages derived from rats with chronic obstructive pulmonary disease examined using western blotting. Herbal group, Quanzhenyiqitang-treated; WM, Western medicine (aminophylline)-treated; HDAC2, histone deacetylase 2.

Levels of caspase-8, caspase-9 and HDAC2 protein expression were significantly higher in AMs in the Quanzhenyiqitang-treated serum group compared with the other three groups (Figs. 5 and 6). The results indicated that Quanzhenyiqitang was capable of inducing apoptosis of AMs derived from rats with COPD by regulating the expression of caspase-8 and caspase-9, and that Quanzhenyiqitang improved the status of the inflammation in COPD by regulating HDAC2.

## Discussion

The pathogenesis of COPD is complicated and usually associated with apoptosis, oxidative stress and imbalances between protease and antiprotease activity, as well as tissue destruction and reconstruction (8). One of the most important clinical manifestations of COPD is chronic inflammation. As a chronic and systemic inflammatory disease, the inflammatory reaction in the lungs may be either the cause or the result of the systemic effects associated with COPD (9). Systemic inflammation may worsen the clinical symptoms of patients, decreasing their activity levels and reducing quality of life, thereby resulting in disease progression and worsening disease prognosis (10).

A laboratory experiment investigating Quanzhenyiqitang indicated that it is capable of improving the tissue structure of damaged organs of multiple systems, including the lungs of rats with COPD (6). In addition, a clinical study demonstrated that Quanzhenyiqitang alleviates the symptoms of COPD and significantly improves the quality of life of patients (11). In the present study, it was hypothesized that Quanzhenyiqitang has therapeutic effects in COPD by controlling inflammation.

The primary characteristics of inflammation in COPD are infiltration of AMs and neutrophils (12). Senior (8) studied lung tissue in patients with COPD and observed that only ~0.3% of AMs underwent apoptosis, demonstrating that the level of apoptosis of AMs in the lungs of patients with COPD was significantly reduced. Furthermore, the reduced rate of apoptosis contributed to the persistence and progression of inflammation in the COPD airway (13).

The HDAC protein is capable of causing DNA condensation, reducing gene transcription and controlling inflammation (14). HDAC2 has an important function in the process of inflammatory reactions in COPD (15). Studies have indicated that the low levels of HDAC activity in bronchial biopsies and AMs of patients with COPD are closely associated with HDAC2 (16), and the degree of decline in HDAC2 activity is closely correlated with the intensity of inflammation (17). Furthermore, the reduction in HDAC2 expression and activity is specific to COPD. In a study of AMs derived from bronchoalveolar lavage fluid of patients with bronchial asthma reduced HDAC2 activity was not observed (18).

Quanzhenyiqitang-treated serum from rats is capable of significantly increasing caspase-8 and caspase-9 expression, inducing apoptosis of AMs in rats with COPD and reducing the number and activity of AMs through the mitochondrial and death receptor pathways on the cell surface (19,20). Thus, it has a significant function in the prevention and control of COPD. Quanzhenyiqitang-treated serum significantly increased HDAC2 activity in AMs from rats with COPD; thus, the dynamic balance between histone acetyltransferases (HATs) and HDAC was adjusted and inflammation was ameliorated.

Quanzhenyiqitang has been demonstrated to be a promising therapeutic agent for COPD. The ability of Quanzhenyiqitang to induce apoptosis of inflammatory cells and regulate the dynamic balance of inflammatory factors may represent only one aspect of its therapeutic actions. The mechanisms of its other effects, including improvement of diaphragm muscle function, digestion and immune status, merit further study.
